# Obituary: Professor Claude Martin

**DOI:** 10.1186/s13054-019-2652-x

**Published:** 2019-11-06

**Authors:** Marc Leone, Jean-Louis Vincent

**Affiliations:** 1 Aix Marseille Université, Assistance Publique Hôpitaux de Marseille, Service d’Anesthésie et de Réanimation, Hôpital Nord, Chemin des Bourrely, 13015 Marseille, France; 2 Department of Intensive Care, Erasme Hospital, Université Libre de Bruxelles, Brussels, Belgium

Claude Martin, born in Boulogne-Billancourt, France, in 1949, died at North Hospital, Marseilles, France, on September 2, 2019. Claude qualified from the Marseille School of Medicine in 1979 and was appointed Professor of Anesthesiology and Intensive Care at South Hospital, Marseilles, in 1989. From 1992 to 2015, he chaired the Department of Anesthesiology and Intensive Care of the North Hospital, Marseilles. His life was dedicated to patient care, demanding the best for all patients admitted to our institution, allowing no concessions in terms of the services provided.

Claude was an outstanding researcher, publishing his first peer-reviewed paper in 1987 [1]. He was a pioneer in many fields, notably promoting the use of norepinephrine in septic shock [2, 3]. He also contributed to advances in gas conditioning/humidification in mechanically ventilated patients, antimicrobial stewardship in severe infection, surgical antimicrobial prophylaxis, and sedation. Many, many physicians have been inspired by his 350 published articles, his informative lectures, and his formidable analytical capacity, based on sound reasoning and carefully checked facts.

Claude was a former president of the French Society of Anesthesiology and Intensive Care, as well as other national organizations. He was member of several international societies, chairing different sections and leading various projects within his fields of interest. He served as an editor for many journals, notably *Critical Care*.

Outside his distinguished medical career, Claude was passionate about comics, rock music, and culture. His favorite pastime was traveling. He was a model of kindness, humanity, and humor and, despite repeated and ongoing health care problems, always continued his activities with a smile. He will be remembered by us all for his gentle elegance even in times of difficulty. During his final hours, Claude Martin was surrounded by his wonderful family.
